# Patient-Reported Experiences of Persistent Post–COVID-19 Conditions After Hospital Discharge During the Second and Third Waves of the Pandemic in Switzerland: Cross-Sectional Questionnaire Study

**DOI:** 10.2196/47465

**Published:** 2024-08-28

**Authors:** Nadine Tacchini-Jacquier, Sévrine Monnay, Nicolas Coquoz, Eric Bonvin, Henk Verloo

**Affiliations:** 1 Development of Nursing Practices Unit Valais Hospitals Sion Switzerland; 2 Social Affairs and Human Resources Valais Hospitals Sion Switzerland; 3 Pneumonology Valais Hospitals Sion Switzerland; 4 General Direction Valais Hospitals Sion Switzerland; 5 Department of Nursing School of Health Sciences, University of Applied Sciences and Arts Western Switzerland (HES-SO) Sion Switzerland; 6 Valais Hospitals Sion Switzerland

**Keywords:** patient-reported experience measures, PREMs, long COVID, fatigue, post-traumatic stress disorder, depression, anxiety, SARS-CoV-2 infection, post-COVID, COVID-19, pandemic, hospital discharge, pandemic

## Abstract

**Background:**

Hospitalized patients infected with SARS-CoV-2 should recover within a few weeks. However, even those with mild versions can experience symptoms lasting 4 weeks or longer. These post–COVID-19 condition (PCC) comprise various new, returning, or ongoing symptoms that can last for months or years and cause disability. Few studies have investigated PCC using self-reports from discharged patients infected with SARS-CoV-2 to complement clinical and biomarker studies.

**Objective:**

This study aimed to investigate self-reported, persistent PCC among patients infected with SARS-CoV-2 who were discharged during the second and third waves of the COVID-19 pandemic.

**Methods:**

We designed, pretested, and posted an ad hoc paper questionnaire to all eligible inpatients discharged between October 2020 and April 2021. At 4 months post discharge, we collected data on PCC and scores for the Multidimensional Fatigue Inventory (MFI), the Patient Health Questionnaire-4 (PHQ-4), a Brief Memory Screening Scale (Q3PC), and a posttraumatic stress disorder scale (PCL-5). Descriptive, inferential, and multivariate linear regression statistics assessed PCC symptomatology, associations, and differences regarding sociodemographic characteristics and hospital length of stay (LOS). We examined whether our variables of interest significantly predicted MFI scores.

**Results:**

Of the 1993 valid questionnaires returned, 245 were from discharged patients with SARS-CoV-2 (median age 71, IQR 62.7-77 years). Only 28.2% (69/245) of respondents were symptom-free after 4 months. Women had significantly more persistent PCC symptoms than men (*P*≤.001). Patients with a hospital LOS ≥11 days had more PCC symptoms as well (*P*<.001)—women had more symptoms and longer LOS. No significant differences were found between age groups (18-64, 65-74, and ≥75 years old; *P*=.50) or between intensive care units and other hospitalization units (*P*=.09). Patients self-reported significantly higher PHQ-4 scores during their hospitalization than at 4 months later (*P*<.001). Three-fourth (187/245, 76.4%) of the respondents reported memory loss and concentration disorders (Q3PC). No significant differences in the median MFI score (56, IQR 1-3, range 50-60]) were associated with sociodemographic variables. Patients with a hospital LOS of ≥11 days had a significantly higher median PCL-5 score (*P*<.001). Multivariate linear regression allowed us to calculate that the combination of PHQ-4, Q3PC, and PCL-5 scores, adjusted for age, sex, and LOS (of either ≥11 days [median 2 symptoms, IQR 1-5] or <11 days), did not significantly predict MFI scores (*R*^2^=0.09; *F*_4,7_ =1.5; *P*=.22; adjusted *R*^2^=0.06).

**Conclusions:**

The majority of inpatients infected with SARS-CoV-2 presented with PCC 4 months after discharge, with complex clinical pictures. Only one-third of them were symptom-free during that time. Based on our findings, MFI scores were not directly related to self-reported depression, anxiety, or posttraumatic scores adjusted for age, sex, or LOS. Further research is needed to explore PCC and fatigue based on self-reported health experiences of discharged inpatients infected with SARS-CoV-2.

## Introduction

During the second and third waves of the COVID-19 pandemic, health care systems focused on dispensing the best available care and preventing the oversaturation of health care services [1-3]. Most health care services marshaled their resources to manage successive waves of hospital admissions and intensive care cases, prioritizing vaccination efforts to protect as many people as possible from severe cases of COVID-19 infection [4]. Given the widespread and multifarious nature of the post–COVID-19 condition (PCC) experienced by populations infected by SARS-CoV-2, coupled with shortcomings in understanding viral-onset illnesses, it is not surprising that there have been few standardized follow-up assessments of the functioning, disability, and health of the patients [5-7]. Patients were sometimes discharged without plans for rehabilitation or any recording of their chronic post–COVID-19 symptoms [3]. Meanwhile, SARS-CoV-2 infected a significant proportion of Switzerland’s population (over 10%), dramatically increasing the number of pneumonia cases, multiorgan failure, and associated risk factors for severe disease and death; less is known about the potential long-term complications of SARS-CoV-2 infection [8]. The World Health Organization defined three criteria for the diagnosis of a PCC: (1) a positive antigenic or serological test for the SARS-CoV-2 virus or, despite a negative test during an acute phase of illness, either a chest computed tomography scan indicative of acute SARS-CoV-2 infection or a typical presentation of it; (2) the presence of symptoms lasting more than 2 months after the onset of symptoms or the acute phase of the disease; and (3) the absence of other reasons or diagnoses that may explain these symptoms [9].

A recent study found that 7 months after COVID-19 onset, 45% of patients had not returned to their previous level of work participation and continued to have a significant symptom burden [10]. A systematic review by Alkodaymi et al [11] examining the enduring signs and symptoms of COVID-19 infection reported pulmonary sequelae, neurological disorders, impaired concentration, generalized anxiety disorder, impairments to functional mobility, fatigue, muscle weakness, and constitutional symptoms—half of the patients included had a PCC lasting more than 6 months. A systematic review and meta-analysis of COVID-19’s long-term effects by O’Mahoney et al [12] reported that at least 45% of COVID-19 survivors were experiencing at least 1 unresolved symptom after 4 months. Fatigue was the most persistent symptom, with a prevalence among hospitalized, nonhospitalized, and mixed-patient cohorts of 28.4%, 34.8%, and 25.2%, respectively [12]. Fatigue could be due to the excessive respiratory efforts related to the respiratory complications of a SARS-CoV-2 infection [13]. However, the lack of a gold-standard scale for assessing fatigue, as well as the subjective nature of this symptom, makes it a poorly evaluated condition. Fatigue is observed in many medical conditions, including cancer, neurodegenerative disorders, rheumatological diseases, and heart failure, but it can also be an isolated symptom with unknown underlying causes, as seen in chronic fatigue syndrome [14]. Other studies have reported that pulmonary abnormalities, including radiological abnormalities and impaired pulmonary function, persist as PCC for months after hospital discharge [3,8,15]. Studies by Xu et al [16] and Brola and Wilski [17] reported stroke, encephalitis, seizures, and conditions, including major mood swings and brain fog, months after the initial onset of a SARS-CoV-2 infection. In addition, COVID-19 has been associated with extending the emotional and behavioral issues surrounding posttraumatic stress disorder (PTSD) [10]. Individuals recovering from COVID-19 infection may be at a greater risk of depression, anxiety, PTSD, and substance use disorder [18-20]. Considering the total number of COVID-19 cases worldwide, the combined effects of this disease have the potential to lead to many different PCC [21]. Between 10% and 20% of people who contracted COVID-19 infection experienced persistent symptoms lasting weeks, months, and even up to 2 years after their infection [12].

This paper emphasizes that PCC should not be limited to biological health markers but should also include self-reported everyday functioning after an infection. In addition, experiences of any signs or symptoms should also be given attention.

Our guiding research questions were the following: (1) What are the persistent symptoms of PCC among inpatients or respondents infected with SARS-CoV-2 4 months after discharge? (2) How do these patients describe the severity of fatigue, depression, anxiety, memory loss, and PTSD?

This study explored the following neutral hypotheses: (1) there is no significant difference between men and women, age groups, hospitalization wards, and lengths of stay (LOS) in self-reported scores for fatigue, depression, anxiety, memory loss, and PTSD at 4 months; and (2) self-reported fatigue scores cannot significantly be explained by the combination of self-reported persistent PCC symptoms, depression, anxiety, memory loss, or PTSD scores.

## Methods

### Design, Research Population, Setting, and Recruitment

A patient-reported experience measures (PREMs) survey was conducted among all the inpatients discharged from the Valais Hospitals between October 14, 2020, and April 22, 2021. The survey aimed to collect data about their hospital experiences and, particularly, any residual symptoms at 4 months post discharge, among patients infected with SARS-CoV-2 during the second and third waves of the COVID-19 pandemic. The Valais Hospitals are a multisite public hospital that recorded more than 40,000 hospitalizations and more than 650,000 ambulatory visits in 2022 [22].

### Study Framework

Based on the principles of patient and public involvement, PREMs of health care delivery have recently become an essential component for recording overall health care system performance [22,23]. PREMs are directly related to the Institute of Healthcare Improvement’s quintuple aim concept [24], whose key transformative health care objectives are improving patients’ experiences, attaining better health outcomes, boosting clinician well-being, lowering costs, and ensuring health equity. This paper reports on the health symptoms experienced by inpatients infected with SARS-CoV-2, 4 months after their discharge during the second and third waves of the COVID-19 pandemic [25].

### Data Collection Instrument

In the absence of a standardized, validated tool for collecting data on PCC, the research team designed a self-reporting questionnaire based on a literature review of PREMs concepts and the epidemiology and consequences of PCC and then pretested it with 4 patients (Multimedia Appendix 1) [26,27]. The paper questionnaire was posted to all eligible patients at 4 months post discharge and included a prepaid envelope for its return. Besides concepts involving PCC, the questionnaire investigated discharged patients’ health, fatigue, posttraumatic stress, cognitive impairments, and other remaining symptoms reported by the participants.

### PCC

#### Health Symptoms After a SARS-CoV-2 Infection

The study investigated the self-reported physical and mental health symptoms of inpatients infected with SARS-CoV-2 who were discharged home. Respondents were given a list of health conditions to indicate whether they had experienced them or not [28-31]. These included persistent weight loss, loss of sense of smell, loss of sense of taste, fever, cold, sore throat, sensations of burning or tingling in upper and lower limbs, persistent paresthesia in the hands or feet, a mobility disorder in one of the limbs, shortness of breath at rest and during daily activities, daily coughing, pain or discomfort in the chest area, hair loss, headaches, muscle aches, the need for home care since leaving hospital, fatigue, and other health conditions reported by the responder.

#### Multidimensional Fatigue Inventory

The Multidimensional Fatigue Inventory (MFI) is a self-administered questionnaire assessing different aspects of fatigue, which are general fatigue, mental fatigue, decreased activity, and motivation [32]. The explored items of the MFI scale are reported in Multimedia Appendix 2. Validated mainly for situations involving cancer, in both French and German, and with a Cronbach for internal consistency of 0.84, this Likert-like scale has possible responses ranging from 1 (completely disagree) to 5 (completely agree). The higher the total score, ranging from 20 to 100, the greater the fatigue. No cutoff points or classifications have been documented using the original scale. Fatigue is a particularly interesting health condition because it is the most prevalent symptom in clinical studies involving PCC and has been explored as a dependent variable in multivariate linear regression analysis [12,18].

#### Brief Memory Screening Scale

The Brief Memory Screening Scale (Q3PC) self-reporting memory scale was used to explore memory loss and attention difficulties among respondents infected with SARS-CoV-2. They were asked the following questions: (1) Do you experience frequent memory loss? (2) Do you feel that you are slower when reasoning, planning activities, or solving problems? (3) Do you have difficulties paying attention? For each question, the response options were 0 (never), 1 (rarely), 2 (sometimes), 3 (often), and 4 (very often) [33]. The higher the score, the worse the participants experienced memory and attention difficulties. The Q3PC demonstrated good psychometric properties, with a Cronbach coefficient of 0.72 [34].

#### Patient Health Questionnaire-4

The 4-item, composite, self-reported Patient Health Questionnaire-4 (PHQ-4) was used to assess anxiety. It was built from the Generalized Anxiety Disorder Scale (GAD-2) and 2 questions identifying a depressive state from the PHQ-2 scale. The 2 items exploring depression are validated based on the *DSM-IV* (*Diagnostic and Statistical Manual of Mental Disorders* [Fourth Edition]) diagnostic criteria for depression, including depressed mood and lack of interest [35]. The 2 GAD-2 questions investigate feelings of nervousness and anxiety and the ability to control one’s worries. The PHQ-4 and GAD-2 are scored 0 (never), 1 (some days), 2 (>50% of days), and 3 (almost every day), with total possible scores of 0-12 for the PHQ-4 and 0-6 for the GAD-2 scale. The PHQ-4 was assessed at baseline during hospitalization and 4 months after discharge. The questionnaire has good psychometric properties, with a Cronbach of 0.78.

#### Posttraumatic Stress Disorder Checklist-5

The PTSD scale (PCL-5) was developed to identify individuals with and those without PTSD and is based on the *DSM-5* (*Diagnostic and Statistical Manual of Mental Disorders* [Fifth Edition]). The checklist includes 20 self-administered items answered using a symptom severity rating ranging from 0 (not at all) to 4 (extremely). Total PCL-5 scores range from 0 (indicating no symptoms) to 80 (indicating very severe symptoms). The cut-off score between “no pathological PTSD symptoms” and “pathological PTSD symptoms” has been estimated to be around 30 to 33—the recommended threshold for a diagnosis of PTSD is 33 points, and a score >30 requires additional investigations [36]. The scale has been translated, culturally adapted, and validated in French, showing excellent internal consistency (Cronbach =0.94) and a test-retest reliability of =0.89 [37]. Forte et al [38] validated the Italian version of PCL-5 during the COVID-19 pandemic, demonstrating the excellent internal consistency of its items (Cronbach =0.94).

### Respondents’ Sociodemographic and Hospital Trajectory Data

In total, 7 closed questions were used to ask participants about their sociodemographic data (eg, sex, age, marital status, and educational level) and hospital trajectory as a patient.

### Data Collection Procedure

**Figure 1:**
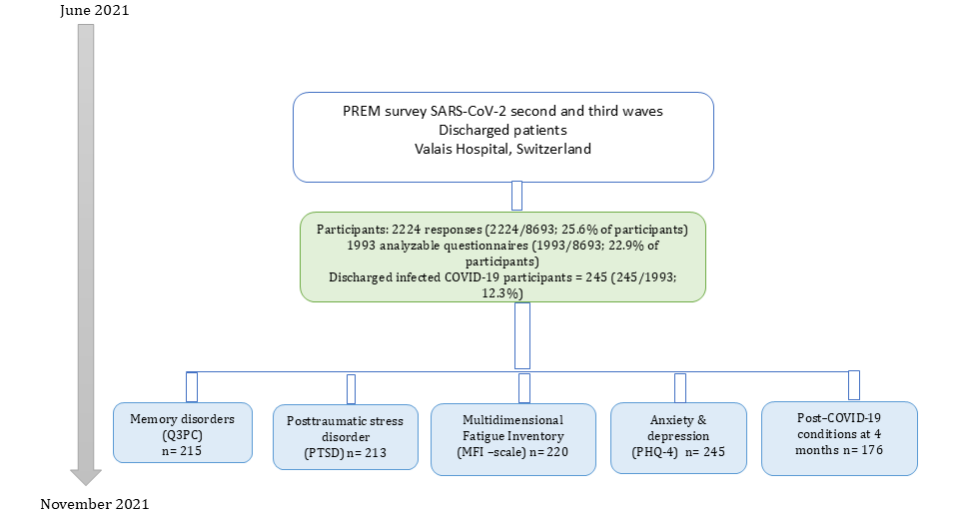
Data collection strategy for post-COVID conditions during second and third waves of COVID-19 in Switzerland, 2020-2021. PHQ: Patient Health Questionnaire; PREMs: patient-reported experience measures; Q3PC: Brief Memory Screening Scale.

Following ethics approval by the Human Research Ethics Committee of the Canton of Vaud (2021-01263), the data science warehouse of the Valais Hospital provided the contact details of all the adult inpatients (18 years and older) discharged alive to their home or a nursing home, between June 21 and November 13, 2021. Eligible patients received a letter by post, including the PREMs questionnaire and an invitation to participate in the survey by completing the attached paper questionnaire. An information sheet explained the background of the study, the data sought, and our participant data protection strategy (Multimedia Appendix 1). Anonymously completing the paper questionnaire and returning it in the prepaid envelope provided was considered a proxy for the informed consent of the participants. A reminder was sent out 4 weeks later (Figure 1).

### Statistical Analysis

#### Overview

Data of the participants were anonymized and good research practices for this type of study were respected, as per the Declaration of Helsinki [39]. Data from the self-reported questionnaires were extracted into an Excel spreadsheet (Microsoft Corporation), cleaned, and imported into IBM SPSS software (version 28.0; IBM Corp), for analyses.

#### Power

With a margin of error (.05), a power of 1 error probability of .80, and an effect size of .5, the total sample size was estimated to be 242 participants. However, a statistically significant sample size might not need to be as large in our PREMs survey because it examines patient experiences with their regular care. Our survey extracted valuable information from respondents about their hospitalization experiences and persistent PCC [40].

#### Data Exclusion

We analyzed the number of responses and missing values for each variable and reported them in Tables 1-5 and Table S1 in Multimedia Appendix 2 (n=answers) [41].

#### Statistics

Descriptive statistics for the population included frequencies, distributions, and leading trends. Parametric properties were analyzed for the normality of their distributions and the equality of their variances. Nonparametric tests were performed for variables with nonnormal distributions describing scores and health conditions of respondents infected with SARS-CoV-2. To test our hypotheses, we computed chi-square statistics for the categorical variables in the contingency tables. Data collected using Likert scales were analyzed using descriptive and inferential statistics and Mann-Whitney and Kruskal-Wallis tests were applied. Because of some extreme outliers, hospital LOS was recorded as a dichotomous variable of 1-11 days or >11 days, based on the median patient LOS of 11 (IQR 5-16.5) days [42-44]. Associations were calculated using the Spearman rank correlation between MFI scores and the number of self-reported PCC and sociodemographic characteristics. We computed a linear multivariate regression model to explore the relationships between MFI scores and the independent variables of PHQ-4, Q3PC, and PCL-5 scores of the patients. The model estimated the net impact of each predictor, assuming other factors remained constant, providing predictions for the entire sample rather than just specific individuals. We computed the internal consistencies of the PHQ-4, Q3PC, and PCL-5 scales using Cronbach coefficients. Values are ≤1, with values ≥0.7 being generally considered “acceptable” [45]. The results were considered statistically significant when *P*<.05. All *P* values were based on 2-tailed tests, and a biostatistician supervised and reviewed all the analyses.

### Ethical Considerations

This study’s research protocol was approved by the Valais Hospitals, the HES-SO Valais-Wallis, Sion, Valais, and the Human Research Ethics Committee of the Canton of Vaud. All our research was carried out in accordance with relevant methodological guidelines and regulations. The Human Research Ethics Committee of the Canton of Vaud (2021-01263) authorized the survey and the extraction of the population-based cohort’s data from administrative, electronic patient records in the hospital’s patient register. Informed consent was obtained from all the participants or their legal representative or representatives. Furthermore, patients and relatives who completed the paper questionnaire and returned it in the prepaid envelope provided were considered to have given their consent to participate in the study.

## Results

### Overview

Of 8693 eligible respondents hospitalized during the second and third waves of the COVID-19 pandemic, between October 2020 and April 2021, a total of 1993 returned valid questionnaires (with >50% of questions completed), representing 89.6% (1993/2224) of the questionnaires returned (n=2224; Figure 1). A total of 245 (245/1993, 12.3%) of those respondents had a confirmed COVID-19 infection, a positive test for SARS-CoV-2, and had been hospitalized for acute COVID-19 symptoms.

### Sociodemographic Characteristics of the Sample

The median age of the infected sample was 71 (IQR 62.7-77) years, with more men participating than women. Most of the respondents were married and had attained education up to the vocational diploma level. The median hospital LOS was 11 (IQR 5-16.5) days. Table S1 in Multimedia Appendix 2 shows the sociodemographic characteristics of the respondents.

### Persistent Symptoms 4 Months After COVID-19 Infection

A total of 69 respondents infected with COVID-19 reported being symptom-free at 4 months. Altogether, 176 respondents infected with COVID-19 reported 643 symptoms, with a median of 3 (IQR 1-3, range 2-11). Overall, 19 respondents reported clinical pictures involving multiple clustered comorbidities or symptoms, such as problems breathing, concentrating, hearing, and sleeping, kidney failure, lack of strength, hallucinations, gastric problems, anxiety, memory and balance problems, and joint pains. The top 3 persistent symptoms were breathing difficulties during physical effort, muscle pain, and shortness of breath at rest (Table 1). Women had significantly more persistent PCC (3 symptoms) than men (2 symptoms). Patients with hospital LOS ≥11 days (2 symptoms) had more persistent PCC too (vs <11 days LOS with 1 symptom). No significant differences were found between age groups or between intensive care units (ICUs) and other hospitalization units. Significant differences in persistent PCC were found between women (3 symptoms) and men (2 symptoms; *P*=.003), depending on hospital LOS of either ≥11 days (2 symptoms) or <11 days (1 symptom; *P*<.001). No significant differences were found between age groups or hospitalization units. The second section of Table 1 presents the distribution of persistent PCC at 4 months, as reported by the respondents.

**Table 1:** Persistent symptoms 4 months after SARS-CoV-2 infection in the Valais Hospitals during the second and third waves of the COVID-19 pandemic in 2020 and 2021.

Variables		Median (IQR 1-3)^a^	*P* value^b^	
**Gender (n=245)**				<.001
	Men (n=145)	2 (0-2)		
	Women (n=100)	3 (1-5)		
**Age category (years; n=242)**				.50
	18-64 (n=71)	2 (1-4)		
	65-74 (n=84)	2 (1-4)		
	75 and older (n=87)	2 (1-5)		
**Hospitalization unit (n=245)**				.09
	ICU^c^ (n=41)	2 (1-5)		
	Other units (n=204)	2 (1-4)		
**Length of stay (days; n=210)**				<.001
	<11 (n=154)	1 (0-4)		
	≥11 (n=56)	2 (1-5)		
**Persisting PCC^d^ symptoms after 4 months, n (%)**				
	Breathing difficulties when active	104 (16.2)	—^e^	
	Muscle pain	77 (12.0)	—	
	Breathing difficulties at rest	56 (8.7)	—	
	Daily coughing	45 (7.0)	—	
	Sensory disorder in hands or feet	40 (6.2)	—	
	Hair loss	35 (5.4)	—	
	Headaches	34 (5.3)	—	
	Neuropathic pain in limbs	30 (4.7)	—	
	Need for home care since hospital discharge	29 (4.5)	—	
	Pain or discomfort in the chest area	28 (4.4)	—	
	Cold	26 (4.0)	—	
	Mobility disorder in a limb	25 (3.9)	—	
	Loss of sense of smell	24 (3.7)	—	
	Loss of sense of taste	23 (3.6)	—	
	Sore throat	18 (2.8)	—	
	Fever	16 (2.5)	—	
	Continuing weight loss	13 (2.0)	—	
	Other symptoms^f^	20 (3.1)	—	

^a^IQR 1-3: interquartile 25%-75%.

^b^Chi-square test.

^c^ICU: intensive care unit.

^d^PCC: post–COVID-19 condition.

^e^Not applicable.

^f^Other self-reported symptoms: joint pain (3/643, 1.2%), balance disorder (2/643, 0.8%), sleep disorder (2/643, 0.8%), memory impairment (2/643, 0.8%), renal decompensation (2/643, 0.8%), hearing loss (1/643, 0.4%), anxiety (1/643, 0.4%), gastric problems (1/643, 0.4%), hallucination (1/643, 0.4%), lack of strength (1/643, 0.4%), concentration disorder (1/643, 0.4%), and pneumonia (1/643, 0.4%).

### Multidimensional Fatigue Inventory

The overall median MFI score among all respondents was 56 (IQR 1-3, range 50-60), showing that most respondents reported moderate to high MFI scores. No significant differences were found between MFI scores and sociodemographic, hospitalization unit, and LOS variables of the patients (Table 2). MFI scale scores of our sample had an internal consistency coefficient Cronbach of 0.45, indicating a low level of consistency [46]. Tables S2 and S3 in Multimedia Appendix 2 present the detailed results.

**Table 2:** Distribution of respondents’ Multidimensional Fatigue Inventory scores based on age, sex, hospitalization unit, and length of stay in the Valais Hospitals during the second and third waves of the COVID-19 pandemic in 2020 and 2021.

Variables		Median (IQR 1-3)^a^	*P* value
**Gender (n=216)**			.36^b^
	Women (n=91)	55 (48-60)	
	Men (n=125)	56 (51-59)	
**Age category (years; n=220)**			.12^c^
	18-64 (n=64)	56.5 (50.2-63)	
	65-74 (n=79)	56 (52-59)	
	75 and older (n=77)	54 (48.5-59)	
**Hospitalization unit (n=220)**			.20^b^
	ICU (n=36)	54 (49.2-57)	
	Other units (n=184)	56 (50.2-60)	
**Length of stay (days; n=219)**			.71^b^
	<11 (n=104)	56 (50-59)	
	≥11 (n=115)	55 (50-60)	

^a^IQR 1-3: interquartile 25%-75%.

^b^Mann-Whitney *U* test.

^c^Kruskal-Wallis test.

### Memory, Concentration, and Attention Disorders: Brief Memory Screening Scale

In total, 40 COVID-19 respondents reported enduring memory loss often to very often, with 109 and 62 declaring mild and no memory loss, respectively. Altogether, 34 respondents reported feeling slow when reasoning through daily problems, 102 stated they rarely or sometimes experienced slowness, and 75 reported never feeling slow in daily reasoning. Overall, 33 respondents reported often or very often having difficulty concentrating, 82 stated they rarely or sometimes experienced concentration problems, and 100 reported no concentration problems. The majority of COVID-19 respondents (n=166) reported one or more disorders on the Q3PC scale, with an overall median of 3 (IQR 1-3, range 1-6) positive responses across the whole group of 245 participants. Considering the cut-off point of ≥1 positive question, 166 respondents present memory loss disorders with a median score of 3 (IQR 1-3, range 1-6). No significant differences were found related to sex, age, or hospital trajectory. On the contrary, a significant difference was found regarding hospital LOS, with patients hospitalized for ≥11 days having higher Q3PC scale scores (Table 3). The Q3PC scale’s internal consistency demonstrated an excellent Cronbach coefficient of 0.89 [46]. Tables S4 and S5 in Multimedia Appendix 2 present the detailed results.

**Table 3:** Distribution of concentration, attention, and memory disorder scores on the Brief Memory Screening Scale in the Valais Hospitals during the second and third waves of the COVID-19 pandemic in 2020 and 2021.

Variables		Median (IQR 1-3)^a^	*P* value	
**Gender (n=213)**				.97^b^
	Women (n=90)	3.9 (1-6)		
	Men (n=123)	3.8 (1-6)		
**Age category (years; n=217)**				.07^c^
	18-64 (n=64)	4.5 (1.2-7)		
	65-74 (n=78)	3 (0-6)		
	75 and older (n=75)	3 (1-6)		
**Hospitalization unit (n=217)**				.20^b^
	ICU^d^ (n=36)	3 (3-6)		
	Other units (n=181)	4 (1-6)		
**Length of stay (days; n=216)**				.03^b^
	<11 (n=103)	3 (1-5)		
	≥11 (n=113)	4 (1-6)		

^a^IQR 1-3: interquartile 25%-75%.

^b^Mann-Whitney *U* test.

^c^Kruskal-Wallis test.

^d^ICU: intensive care unit.

### Depression and Anxiety Disorders: PHQ-4

After 4 months, 14 respondents still had an elevated score for symptomatic health issues and 16 presented a moderate score for symptomatic mental health impairment. Significant differences between men (median 0, IQR 0-4) and women (median 1, IQR 0-8; *P*=.03), between patients in ICU (median 1, IQR 1.5-8) and patients in other hospital units (median 1, IQR 0-4; *P*=.04), and between hospital LOS ≥11 days (median 1, IQR 0-4) and <11 days (median 1, IQR 0-6; *P*=.03), were found 4 months after a SARS-CoV-2 infection. No significant differences were found between the age groups (*P*=.82; Table 4). The internal consistency of the PHQ-4 scale showed an excellent Cronbach of 0.88 [46]. Tables S6 and S7 in Multimedia Appendix 2 present the detailed results.

**Table 4:** Distribution of the Patient Health Questionnaire-4 scores among respondents at 4 months post SARS-CoV-2 infection in the Valais Hospitals during the second and third waves of the COVID-19 pandemic in 2020 and 2021.

Variables		Median (IQR 1-3)^a^	*P* value	
**Gender (n=241)**				.03^b^
	Women (n=100)	1 (0-4)		
	Men (n=141)	0 (0-3)		
**Age category (years; n=238)**				.82^c^
	18-64 (n=64)	1 (0-3)		
	65-74 (n=87)	0 (0-2)		
	75 and older (n=87)	1 (0-3)		
**Hospitalization unit (n=245)**				.04^b^
	ICU^d^ (n=41)	2 (0-4)		
	Other units (n=204)	0 (0-3)		
**Length of stay (days; n=244)**				.03^b^
	<11 (n=120)	1 (0-2)		
	≥11 (n=124)	1 (0-4)		

^a^IQR 1-3: interquartile 25%-75%.

^b^Mann-Whitney *U* test.

^c^Kruskal-Wallis test.

^d^ICU: intensive care unit.

### PTSD Assessment

The overall median PCL-5 score among the respondents infected with SARS-CoV-2 was 12 (IQR 1-3, range 4-22). Significant differences were found between the respondents’ hospital LOS of either ≥11 days or <11 days, with higher PCL-5 scores among respondents with longer LOS (*P*=.01). No differences were found regarding sex, between patients in ICU and patients in other hospitalization units, or between the age groups (Table 5). The internal consistency of the PCL-5 scale showed an excellent Cronbach coefficient of 0.95 [46]. Tables S8 and S9 in Multimedia Appendix 2 present the detailed results.

**Table 5:** Distribution of posttraumatic stress disorder scale scores according to sex, age category, hospitalization unit, and length of stay in the Valais Hospitals during the second and third waves of the COVID-19 pandemic in 2020 and 2021.

Variables			Median (IQR 1-3)^a^	*P* value	
**Gender (n=211)**					.35^b^
	Women (n=87)	17.5 (4-26)			
	Men (n=124)	14.7 (4-21.7)			
**Age category (years; n=215)**					.06^c^
	18-64 (n=64)	13.5 (6-31.7)			
	65-74 (n=78)	13.2 (3-17.2)			
	75 and older (n=73)	16.2 (3.5-25.5)			
**Hospitalization unit (n=215)**					.18^b^
	ICU^d^ (n=36)	20.5 (5-31.5)			
	Other units (n=179)	14.7 (4-21)			
**Length of stay (days; n=214)**					.01^b^
	<11 (n=105)	12.7 (4-19.5)			
	≥11 (n=109)	18.7 (4-30)			

^a^IQR 1-3 = interquartile 25%–75%.

^b^Mann-Whitney *U* test.

^c^Kruskal-Wallis test.

^d^ICU: intensive care unit.

### Associations Between MFI Scores and Numbers of Post–COVID-19 Symptoms

We computed a Spearman rank correlation between MFI scores and the number of persistent PCC symptoms in patients, but no significant associations were found (_s_=0.06; *P*=.36).

### Fatigue Score Predictivity of Persistent PCC Symptoms and Health Questionnaire, Memory Disorder, and Posttraumatic Stress Disorder Scores

A multivariate linear regression was conducted to examine how well the combination of numbers of persistent PCC symptoms, along with the PHQ-4, Q3PC, and PCL-5 scores, predicted MFI scores. When adjusted for sex, age, and hospital LOS, they did not significantly predict MFI scores, with an *R*^2^=.09 (*P*=.22) and an adjusted *R*^2^=.06. According to Cohen, this was a low effect [47]. The weights and determining values, presented in Table S10 in Multimedia Appendix 2, indicate that the Q3PC and PCL-5 scores contributed the most to predicting the MFI scores.

## Discussion

### Principal Findings

The majority of patients infected with and hospitalized for SARS-CoV-2 presented with persistent PCC, often with complex clinical pictures and a wide range of symptoms. Less than a third of discharged infected patients were symptom-free after 4 months.

The PREMs concept is recognized as a valuable method of collecting patients’ self-reported data. It helps assess health care system performance using relevant concepts and mostly self-reporting tools [23]. By giving a voice to health care end users, we consider our self-reported empirical data collection to be a relevant scientific approach, coherent with the methods developed by the Institute for Healthcare Improvement [48,49]. PREMs are now widely recognized as a sensitive method for reporting on accessibility, communication, continuity, and health care system confidence [50].

Our sample of patients with persistent PCC comprised more men than women, but our results showed that women reported significantly higher numbers of PCC symptoms than men. Indeed, sex differences in outcomes were reported during earlier COVID-19 outbreaks, so the differences in this study are unsurprising. Currently, the sex-related long-term consequences of PCC remain poorly studied [51]. However, the studies by Tran et al [52] (85% women) and Bai et al [53] were consistent with our results, showing that women had significantly higher numbers of PCC symptoms than men.

Our findings suggest that self-reported depression, anxiety, PTSD, and health impairments did not significantly predict the MFI scores reported by our respondents. We postulated numerous ideas about our results. First, the use of self-reporting questionnaires played a central role in the assessment of signs and symptoms. However, self-reported questionnaires can be a barrier to producing reliable answers from participants with the same clinical presentation [54]. Consequently, one disadvantage of our self-reporting questionnaire could be invalid answers. Respondents may not answer truthfully about such sensitive issues as depression or anxiety because of a social desirability bias. Another issue could be a response bias, which is an individual’s tendency to respond in a certain way regardless of the question, known as either acquiescent response bias (ticking yes responses) or nonacquiescent response bias (ticking no responses). Respondents with elevated levels of depression, anxiety, or PTSD may have underreported certain categories of symptoms compatible with COVID-19 infection. This could have important effects on how well certain variables are able to predict MFI scores for persistent PCC among respondents infected with SARS-CoV-2. Another potential problem might be how clear or understandable items were for discharged older adult patients, which raises the risk of questions being interpreted differently. Moreover, highly structured questionnaires may induce participants to answer in ways that do not match their true views [55]. Another explanation could be that the presence of cognitive impairment, depressive disorders, or fatigue influences the answers of the patients. A growing number of investigations on PCC have used self-reporting questionnaires that were not specifically developed for PCC but rather for respiratory conditions (Medical Research Council Dyspnea Scale), anxiety disorders (GAD assessment), and depression (PHQ-4) [56]. The development of validated tools specifically designed to assess PCC would enhance comparability and epidemiological robustness, as recommended by Bull et al [22] and Beattie et al [57]; however, this development is still in progress.

Finally, we hypothesize the presence of floor or ceiling effects in the ad hoc questionnaire [58,59].

### Comparison With Previous Work

Collecting data on PCC at 4 months was in line with existing studies exploring persistent PCC. The systematic review conducted by O’Mahoney et al [12] included 194 studies of PCC among hospitalized and nonhospitalized patients that reported assessments from 28 to 387 days after COVID-19 infection, with an average follow-up of 124 days.

Our findings about persistent PCC symptoms were consistent with existing literature on fatigue, pain, memory impairments, breathlessness, and psychological and distress disorders. O’Mahoney et al [12] mentioned that the most important prevalent PCC symptoms were fatigue (28.4%), pain or discomfort (27.9%), impaired sleep (23.5%), breathlessness (22.6%), and impaired memory (22.3%), corroborating the systematic review by Salari et al [60], who also mentioned the appearance of a fatigue syndrome 4 weeks after the onset of COVID-19 symptoms. Moreover, numerous studies have reported persistent fatigue to be a major PCC symptom—despite patients receiving medical and health care, their severe fatigue showed little or no improvement 3 to 6 months after treatment, and worse, PCC fatigue may persist for more than 6 months [12,60,61].

Multiple authors have reported that long COVID can present a similar clinical picture to chronic fatigue syndrome or other persisting illnesses [62-64]. Our multivariate linear regressions tested whether MFI scores could be significantly predicted by other symptoms experienced by patients and related to fatigue, such as depression, anxiety, somatic health, or posttraumatic disorders. Recent research has reported a relationship between long COVID fatigue, chronic fatigue syndrome, physical deconditioning, and mental and somatic disorders [63-65].

Neurological symptoms reported by our respondents, in the form of cognitive and attention impairments, corroborated with Guo et al [66], who reported that SARS-CoV-2 infection affected multiple patients with neurological symptoms and neural damage, affecting between 10% and 25% of patients infected with SARS-CoV-2 with cognitive and attention impairments. Furthermore, Price [67] reported symptoms of cognitive impairment in about 62% of adults with PCC symptoms, compared with 30% among those who had never had post–COVID-19 symptoms.

Multiple studies have reported specific aspects of the hospitalization experience to be associated with long-term psychological difficulties and stress among patients severely affected by COVID-19 [68,69]. However, the physical, psychological, and functional problems of patients with PCC recovering at home must be considered together [70,71]. Research on PTSD and SARS-CoV-2 infections indicated that psychological distress was more severe among groups that had contracted the infection than among other patients with severe illness hospitalized at the same time [20,69]. These studies documented the posthospitalization psychological difficulties that manifested themselves in stress, fear, depression, persistent acute confusion, and disorders based on continuous stressors, such as sleep and memory disorders and attention difficulties [68,72].

Our results revealed the physical and emotional consequences of living with PCC, including stress and mood disorders. The scientific community needs to better understand these health issues, and they need to be more clearly explained to health policy decision-makers. People experiencing PCC symptoms deserve close symptom and biological monitoring using new or existing health care services resources [31,73,74]. What causes PCC symptoms, including chronic fatigue, and why only certain people experience them, still requires further exploration, as recent systematic reviews have noted [51,75]. Recent studies have made it apparent that many patients with COVID-19 experience persistent PCC symptoms, even after the acute infection has been treated. These symptoms may be specific to COVID-19 or secondary symptoms related to hospitalization, including hospitalization in ICU [2,3,73]. Self-reported PHQ-4 scores were in line with the online Swiss Corona Immunitas study, describing self-reported PHQ-4 scores in the more acute phases of COVID-19 infection, among hospitalized and nonhospitalized participants. Indeed, lingering systematic somatic symptoms were associated with higher PHQ-4 scores [76].

Overall, our results indicated that only one-third of our respondents infected with SARS-CoV-2 reported being free of PCC symptoms after 4 months. This was substantially higher than the 10%-20% proportions of PCC sequelae at 4 months mentioned in the reports of the United Kingdom’s National Institute of Health and Care Excellence, its Royal College of General Practitioners and Healthcare Improvement Scotland, and the World Health Organization [73,77,78]. We hypothesize that this difference was due to the severity of the SARS-CoV-2 infections among our hospitalized respondents. However, this was not entirely supported by our data and needs more detailed research data. Health care systems worldwide will face significant pressure on their services, providing care for patients with PCC, including their morbidity and the health care costs of optimally managing those individuals [79,80].

### Limitations

This study had some limitations. The PREMs approach shows the challenges of relying on self-reported questionnaires that may become cognitively burdensome to patients with PCC, failing to comprehensively capture the spectrum of symptoms. Consequently, we cannot directly engage with the underlying biological mechanisms. Furthermore, our study design failed to give any precise estimates of symptom persistence, and it relied on respondent recall 4 months after the initial illness. Furthermore, among those respondents infected by SARS-CoV-2, we had no way of estimating the extent to which apparent PCC might have been the consequences of other illnesses. Finally, we relied on the self-reporting of symptoms rather than objective physiological or cognitive measures. As such, our results should be seen as complementary to, rather than a replacement for, analyses using patients’ electronic health records and other prospective cohort studies.

Nonetheless, our PREMs survey may lack rigor and the accuracy of the information provided cannot be verified. In addition, the results should be interpreted with caution and not be considered generalizable in other regions for patients infected with PCC discharged home.

### Conclusions

This study highlighted that some patients experience persistent clusters of related health issues long after a SARS-CoV-2 infection. Fatigue, cognitive impairments, and breathlessness were the most prevalent symptoms reported, found throughout PCC trajectories of the patients and commonly cited as PCC in numerous other studies [12]. The fatigue they felt could not be properly explained by other potential mental and physical health issues or etiologies in our sample.

Persistent PCC will surely have long-term implications for individuals and society. Given the challenges and negative effects that individuals with PCC must face, more studies conducted using patient-reported experience measures to investigate PCC will bring further insights. One fundamental question requiring further investigation is how the differences in the prevalence of PCC vary according to a range of sociodemographic correlates. The need for a broader understanding of and more information about PCC could be addressed by investigating the lived experiences of patients, as they are ideally placed to provide expert opinions. Our results are relevant to patients, clinicians, and policymakers regarding the long-term outcomes of COVID-19, the need to support appropriate PCC treatment pathways, and the need for future studies aligned with PCC.

## Appendix

Multimedia Appendix 1 Self-reporting questionnaire.

Multimedia Appendix 2 Supplementary File 2.

## Abbreviations

*DSM-IV*: *Diagnostic and Statistical Manual of Mental Disorders (Fourth Edition)*
*DSM-5*: *Diagnostic and Statistical Manual of Mental Disorders (Fifth Edition)*
GAD-2: Generalized Anxiety Disorder-2 Scale
ICU: intensive care unit
LOS: length of stay
MFI: Multidimensional Fatigue Inventory
PCC: post–COVID-19 condition
PCL-5: Posttraumatic Stress Disorder Scale
PHQ-4: Patient Health Questionnaire-4
PREM: patient-reported experience measure
PTSD: posttraumatic stress disorder
Q3PC: Brief Memory Screening Scale

## Data Availability

The data sets generated during and/or analyzed during this study are available from the corresponding author on reasonable request. PREMs data are archived in REDCap (Research Electronic Data Capture) software.
