# Supplementary material for: Patient-Reported Experiences of Persistent Post–COVID-19 Conditions After Hospital Discharge During the Second and Third Waves of the Pandemic in Switzerland: Cross-Sectional Questionnaire Study
Source: JMIR Public Health Surveill. 2024 Aug 28;10:e47465. doi: 10.2196/47465 (PMC11391158; doi:10.2196/47465)
Supplement: Multimedia Appendix 1 [file publichealth_v10i1e47465_app1.pdf]

Hôpital du Valais – Direction générale
Avenue du Grand-Champsec 86, CH-1951 Sion

Direction générale, Case postale 696, CH-1951 Sion

Hôpital du Valais
Spital Wallis

Sion, 28.07 2021

SUPPLEMENTARY FILE 1

Your contacts: Sévrine Monnay / 027 60 36766 / sevrine.monnay@hopitalvs.ch
Nadine Tacchini-Jacquier / 027 60 36752 / nadine.tacchini-jacquier@hopitalvs.ch

Invitation to participate in a questionnaire survey of your lived experiences of your hospitalisation during the second wave of the COVID-19 pandemic

Dear Madame, Dear Sir,

The development of the COVID-19 pandemic's second wave required adaptations from hospitals' medical and nursing staff and from patients and their relations. You were hospitalised at the Hôpital du Valais between 14 October 2020 and 22 April 2021. Although health-care professionals had to show significant resilience during this crisis situation, we would like to know about your emotional, medical and organisational lived experiences of your stay in our institution. Our goal is to improve future care.

To achieve this, we would be very grateful if you could take the time to complete the attached questionnaire and return it to us in the attached stamped, addressed envelope, preferably before **15 November 2021**. The questionnaire is made up of two parts. The first part concerns you directly; the second part concerns the relative (and/or caregiver) who was most involved in your hospitalisation (if such a person exists) so that they can also express themselves.

You are free to respond or not, and there will be no consequences should the questionnaire not be completed. If you return the questionnaire, we will consider that you have consented to participate in this survey. There will be no financial remuneration for your participation. We comply with all the legal data protection requirements mentioned under the Swiss Federal Human Research Act (LRH,2014). All the persons involved in the study are subject to the rules of professional secrecy. Your personal data and any data linked to your health are protected and used in a coded form in order to ensure anonymity. Only the questionnaire data will be used and can be published in an aggregate format where the participants will be unidentifiable.

Nadine Tacchini-Jacquier MSc
Project Leader
Development of Good Practices
Hôpital du Valais

Sévrine Monnay
Social Affairs and
Human Resources
Specialist
Hôpital du Valais

Henk Verloo, PhD
Professor
HES-SO Valais/Wallis

Consultant scientifique We
Hôpital du Valais

thank you for your valuable help, wish you a prompt recovery and send you our best regards.

Annexes: - questionnaire
- prepaid envelope

Survey of your lived experiences as a patient and of those of your family caregivers throughout your hospitalisation during the COVID-19 pandemic.
(Hospitalisation from 14 October 2020 to 22 April 2021)

1. **Personal information** (please complete and tick)
   - Your age: ……….
   - Sex: □ Female □ Male
   - Marital status: □ Married □ Single □ Divorced/Separated □ Widowed
   - What is your highest level of education?
     □ Compulsory schooling □ High school (and/or an apprenticeship)
     □ Higher studies
   - Was a family member (and/or family caregiver) involved throughout your hospitalisation?
     □ Yes □ No
     If yes, and you agree, the questions on page 8 should be filled in by your family caregiver and then returned in the same envelope as your answers.
2. **Date of hospital admission** (day/month): ……….
3. **Date of hospital discharge** (day/month): ……….
4. **Please tick the different hospital departments that you attended during your hospitalisation** (more than one option is possible)
   - □ Emergency department
   - □ Medical care department
   - □ Surgery department
   - □ Psychiatry department
   - □ Gynaecology/maternity department
   - □ Rehabilitation unit
   - □ Continuous care unit
   - □ Intensive care unit
   - □ Others:……………………………..
5. **Were you hospitalised because of an infection by COVID-19?**
   □ Yes □ No
6. **How serious do you think an infection with COVID-19 is?**

Not at all serious	Not very serious	Slightly serious	Serious	Very serious
□	□	□	□	□

7. **How did you perceive the information you received about the COVID-19 pandemic during your hospital stay?** (please tick one box only)
   - □ Totally inadequate
   - □ Inadequate
   - □ Slightly inadequate
   - □ No opinion (neutral)
   - □ Just adequate enough
   - □ Adequate
   - □ Very adequate
8. **How would you rate the communication about the COVID-19 pandemic during your hospital stay?** (please tick one box only)

Poor	Passable	Good	Very good	Excellent
☐	☐	☐	☐	☐

9. **How safe did you feel during your hospital stay?** (please tick a number between 1 and 10 to give your assessment of your feeling of safety, with 10 being completely safe and 1 being completely unsafe)

Completely unsafe — Completely safe

1	2	3	4	5	6	7	8	9	10

10. **The following questions are about the trust you felt in your physicians and nurses throughout your hospital stay during the pandemic.**
Please tick the box which best corresponds to your opinion about each statement.

	Totally disagree	Moderately disagree	No opinion	Moderately agree	Totally agree
a) The health-care staff really care about me as a person					
b) The health-care staff is usually considerate of my needs and makes them a priority.					
c) I have a lot of trust in health-care staff, and I always try to follow their advice.					
d) If health-care staff tell me that this is the way it is, then it must be true.					
e) I trust health-care staff's judgement on my medical care.					
f) I feel that health-care staff do not do everything they should concerning my medical treatments.					
g) Health-care staff are real experts in the management of medical problems like mine.					
h) I trust that health-care staff will tell me if a mistake has been made in my treatment.					
i) I trust that health-care staff will keep the information we discuss confidential.					
**j)** I sometimes distrust health-care staff's opinions and like to get a second opinion. (Note the negative formulation)					

11. **How often did you encounter the following problems during your hospitalisation?**

	Never	Some days	More than half of the days	Almost every day
a) Feeling nervous, anxious or on edge	☐	☐	☐	☐

b) Not being able to stop or control my worries.	☐	☐	☐	☐
c) Having little interest or pleasure in doing things.	☐	☐	☐	☐
d) Feeling demoralised, depressed or desperate.	☐	☐	☐	☐

And how often do you encounter the following problems today?

	Never	Some days	On more than half of the days	Almost every day
e) Feeling nervous, anxious or on edge	☐	☐	☐	☐
f) Not being able to stop or control my worries.	☐	☐	☐	☐
g) Having little interest or pleasure in doing things.	☐	☐	☐	☐
h) Feeling demoralised, depressed or desperate.	☐	☐	☐	☐

12. **Did your health problem, combined with your hospitalisation during a period when the canton of Valais was facing a pandemic, cause you to feel stressed?**
Please respond to the following 10 questions as spontaneously as possible.

During your hospitalisation, how often did you:	Never	Almost never	Sometimes	Fairly often	Very often
1. Feel upset because of something that happened unexpectedly?	☐	☐	☐	☐	☐
2. Get the impression that you were unable to control the important things in your life?	☐	☐	☐	☐	☐
3. Feel nervous or stressed?	☐	☐	☐	☐	☐
4. Feel confident in being able to face up to your problems?	☐	☐	☐	☐	☐
5. Feel that things were going the way you wanted them to?	☐	☐	☐	☐	☐
6. Feel overwhelmed by all of the things that you had to get done?	☐	☐	☐	☐	☐
7. Feel capable of managing the inconveniences you encountered?	☐	☐	☐	☐	☐
8. Feel that you were functioning to the best of your capacities?	☐	☐	☐	☐	☐
9. Feel annoyed because events were outside of your control?	☐	☐	☐	☐	☐
10. Feel that your difficulties were so enormous that you could not control them?	☐	☐	☐	☐	☐

13. **Were your relations able to visit you during your hospitalisation?**

☐ Yes ☐ No

If not, did this affect you? (please tick one answer only)

No opinion	I was not affected	I was slightly affected	I was moderately affected	I was very affected
☐	☐	☐	☐	☐

14. **Are there any people among your entourage who can support you during your illness?**

□ A spouse □ A friend □ Adult children □ Spiritual advisors □ Nobody

□ Others……………….

15. **Were you aware that psychological support was available to you throughout your hospital stay?**

    □ Yes □ No

16. **Did you receive any psychological support throughout your hospitalisation?**

    □ Yes □ No

    If you did benefit from psychological support, were you satisfied with that service? Please circle the most appropriate number

    Not at all satisfied — Extremely satisfied

1	2	3	4	5

17. **Do you think that you still need psychological support today?**

    □ Yes □ No □ I do not know

18. **If you were infected by the coronavirus, do you still have any physical or psychological symptoms associated with that infection?**

    □ No □ Yes

    If yes, please tick off your symptoms listed on pages 7 and 8.

19. **Was there anything missing or lacking from your hospital discharge?**

    □ No □ Yes If yes, please give details:

    …………………………………………………………………………………………………

    …………………………………………………………………………………………………

With the aim of improving the care provided, do you have any other suggestions to offer after your experience in hospital?

…………………………………………………………………………………………………

…………………………………………………………………………………………………

…………………………………………………………………………………………………

If you were not infected by the coronavirus, your section of the questionnaire ends here. You can now transmit **part 2 to your relative**. We thank you very much for your participation.

Hôpital du Valais – Direction générale
Avenue du Grand-Champsec 86, CH-1951 Sion

This list of symptoms is for people who fell ill following their infection with the coronavirus.

Please put a cross next to any symptoms that you have suffered from since your discharge from hospital.

	Continued weight loss		Difficulty breathing, even at rest (shortness of breath)
	Loss of sense of smell		Difficulty breathing when active
	Loss of sense of taste		Daily coughing
	Fever and/or chills		Pain or discomfort in the thorax
	Cold		Hair loss
	Sore throat		Headaches
	Burning sensations or electric-like pains in the limbs		Muscle pain
	Loss of sensation in the hands or feet (persistent paraesthesia)		A need for home care since hospital discharge
	A limb mobility disorder. If yes, which one:……………		Other………………………………………..

Responding to the following statements will help us to understand how you are feeling.
After reading each phrase, please respond by **circling the number** that best suits you. Number 1 signifies that you totally disagree with the statement, and number 5 signifies that you totally agree with it. (MFI20)

Totally disagree				Totally agree
1	2	3	4	5

a) I feel fit	1	2	3	4	5
b) Physically, I do not feel capable of doing much	1	2	3	4	5
c) I feel very active	1	2	3	4	5
d) I want to do lots of nice things	1	2	3	4	5
e) I feel tired	1	2	3	4	5
f) I think that I do lots of things in a day	1	2	3	4	5
g) I am able to concentrate on what I am doing	1	2	3	4	5
h) Physically, I can do a lot (good physical stamina)	1	2	3	4	5
i) I get stressed by the idea of having to do something	1	2	3	4	5
k) I think that I do very little during a day	1	2	3	4	5
l) I feel rested	1	2	3	4	5
m) It takes a lot of effort for me to concentrate	1	2	3	4	5
n) Physically, I feel in poor shape	1	2	3	4	5
o) I have lots of projects	1	2	3	4	5
p) I get tired easily	1	2	3	4	5
q) I do not get much done	1	2	3	4	5
r) I do not feel like doing anything	1	2	3	4	5
s) My thoughts easily wander	1	2	3	4	5

www.hopitalvs.ch
www.spitalvs.ch

t) Physically, I feel in great shape	1	2	3	4	5

Do you have any memory problems? Q3PC Please tick one box on each line.

	No	Rarely	Sometimes	Often	Very often
Do you experience memory loss?					
Do you feel any slowness in your reasoning abilities, in planning your activities or in solving problems?					
Do you have any difficulties concentrating or focusing your attention? (e.g. when following a conversation, reading or watching a TV programme)					

During stressful experiences, such as the COVID-19 pandemic, some people can experience the problems in the list below. To what extent have you experienced these problems during the last month? (PCL-5) Please circle the appropriate number.

	Not at all	A little	Sometimes	Often	Very often
Repeated, distressing and involuntary memories of the stressful experience	0	1	2	3	4
Repeated distressing dreams of the stressful experience	0	1	2	3	4
Suddenly feeling as if the stressful experience was recommencing (as if you were reliving it)	0	1	2	3	4
Feeling distressed or overwhelmed when something reminds you of the experience	0	1	2	3	4
Experiencing a physical reaction when something reminds you of the stressful experience (e.g. a faster heartbeat, trouble breathing, or sweating)	0	1	2	3	4
Avoiding memories, thoughts or feelings associated with the stressful experience (e.g. people, places, conversations, activities, objects or situations)	0	1	2	3	4
Difficulty remembering important things about the stressful experience	0	1	2	3	4
Having negative thoughts about oneself, others or the world (e.g. thoughts such as 'I am bad,' 'there's something seriously wrong with me,' 'I can't trust anybody,' 'the world is a very dangerous place')	0	1	2	3	4
Experiencing intense negative feelings, such as fear, horror, anger, guilt, or shame	0	1	2	3	4
Experiencing intense negative feelings, such as fear, horror, anger, guilt, or shame	0	1	2	3	4
Losing interest in activities that one once enjoyed	0	1	2	3	4
Feeling distant or cut off from others	0	1	2	3	4
Experiencing difficulties having positive feelings (e.g. being incapable of feeling joy or love for one's loved ones)	0	1	2	3	4
Being irritable, having angry outbursts, or acting aggressively	0	1	2	3	4
Taking reckless risks or behaving in ways that might put one in danger	0	1	2	3	4
Being hyper-alert, vigilant or on one's guard	0	1	2	3	4
Being easily startled	0	1	2	3	4
Having difficulties concentrating	0	1	2	3	4
Having difficulties falling or staying asleep	0	1	2	3	4

Part 2

This part of the questionnaire should be filled in by the person who acted as your **close family caregiver** throughout your hospitalisation. If you did not have any family members directly involved during your hospitalisation, please leave this section blank.

1. **Were you able to visit your relation in hospital?**

   ◻ Yes ◻ No

   If not, did this affect you? (please tick one answer only)

No opinion	I was not affected	I was slightly affected	I was moderately affected	I was very affected
◻	◻	◻	◻	◻

2. **How did you perceive the information you received about the COVID-19 pandemic throughout your relation's hospital stay?** (please tick one box only

   - ◻ Totally inadequate
   - ◻ Inadequate
   - ◻ Slightly inadequate
   - ◻ No opinion (neutral)
   - ◻ Just adequate enough
   - ◻ Adequate
   - ◻ Very adequate

3. **How would you rate communication with the staff?** (please tick one box only)

Poor	Passable	Good	Very good	Excellent
◻	◻	◻	◻	◻

4. **As a close family caregiver, how did the hospital staff consider you?** (please tick one box only)

I was not considered at all	I was moderately considered	I was fully considered
◻	◻	◻

5. **How serious do you think an infection with COVID-19 is?** (please tick one box only)?

Not at all serious	Not very serious	Slightly serious	Serious	Very serious
◻	◻	◻	◻	◻

Would you like to add any comments about your experience of your relation's hospitalisation during the pandemic?

....

....

....

We sincerely thank you for taking the time to complete this survey.

Please return this questionnaire in the stamped addressed envelope provided.
