# Supplementary material for: Patient-Reported Experiences of Persistent Post–COVID-19 Conditions After Hospital Discharge During the Second and Third Waves of the Pandemic in Switzerland: Cross-Sectional Questionnaire Study
Source: JMIR Public Health Surveill. 2024 Aug 28;10:e47465. doi: 10.2196/47465 (PMC11391158; doi:10.2196/47465)
Supplement: Multimedia Appendix 2 [file publichealth_v10i1e47465_app2.docx]

**Supplementary File 2**

**Table 1.** Distribution of COVID-19–infected discharged inpatients’ sociodemographic data in Valais Hospitals during the second and third waves of the COVID-19 pandemic in 2020 and 2021.

| Variables | | Respondents infected with SARS-CoV-2 | *P* values^a^ |
| --- | --- | --- | --- |
| **Gender (n=245), n (%)** | | | <.01 |
|  | Men | 145 (59) |  |
|  | Women | 100 (41) |  |
| **Age (years) (n=245)** | | | <.01 |
|  | Median (IQR 1-3)^b^ | 71 (62.7-77) |  |
|  | Age range (min-max) | 31-96 |  |
| **Marital status (n=236), n (%)** | | | <.01 |
|  | Single | 20 (8.5) |  |
|  | Married | 148 (62.7) |  |
|  | Divorced or separated | 23 (9.7) |  |
|  | Widowed | 45 (19.1) |  |
| **Education (n=231), n (%)** | | | <.01 |
|  | Primary school | 77 (33.3) |  |
|  | Vocational diploma | 103 (44.6) |  |
|  | Higher education or university | 51 (22.1) |  |

^a^Chi-square exact test.

^b^IQR 1-3: interquartile 25%-75%.

#### Multidimensional Fatigue Inventory - MFI

The explored items of the MFI scale explored the following items: a) I feel fit, b) I do not feel able to do much physically, c) I feel very active, d) I want to do lots of fun things, e) I feel tired, f) I think I do a lot in a day, g) I am able to concentrate on what I am doing, h) I can do a lot physically (good physical stamina), i) I am stressed about having to do things, j) I think I get very little done in a day, k) I feel rested, l) It takes a lot of effort to concentrate, m) I feel out of shape physically, n) I have lots of plans, o) I get tired easily, p) I do not get much done, q) I do not feel like doing anything, r) My thoughts wander easily, s) I feel in excellent shape physically, and t) I have difficulty concentrating or focusing my attention.

Table 3.1. Additional/detailed data: Distribution of self-reported Multidimensional Fatigue Inventory (MFI) scores after 4-6 months of SARS-CoV-2 infection.

| Items | Median  (IQR 1–3) | Completely disagree (1)  n (%) | Partially disagree  n (%) | Neither agree nor disagree  n (%) | Partially agree  n (%) | Completely agree  n (%) |
| --- | --- | --- | --- | --- | --- | --- |
| 1. (n = 204) | 3 (3–4) | 14 (6.9) | 28 (13.7) | 64 (31.4) | 63 (30.9) | 35 (17.5) |
| 1. (n = 202) | 3 (1–3) | 61 (30.2) | 36 (17.8) | 59 (29.2) | 32 (15.8) | 14 (6.9) |
| 1. (n = 204) | 3 (1–3) | 28 (13.7) | 35 (17.2) | 58 (28.4) | 54 (26.5) | 29 (14.2) |
| 1. (n = 204) | 4 (3–5) | 9 (4.4) | 25 (12.3) | 46 (22.5) | 58 (28.4) | 66 (32.4) |
| 1. (n = 212) | 3 (2–4) | 36 (17.0) | 36 (17) | 57 (26.9) | 42 (19.8) | 41 (19.3) |
| 1. (n = 204) | 3 (2–4) | 17 (8.3) | 39 (19.1) | 60 (29.4) | 57 (27.9) | 31 (15.2) |
| 1. (n = 208) | 4 (3–5) | 15 (7.2) | 23 (11.1) | 47 (2.6) | 61 (29.3) | 62 (29.8) |
| 1. (n = 206) | 3 (2–4) | 33 (16) | 45 (21.8) | 68 (33.0) | 43 (20.9) | 17 (8.3) |
| 1. (n = 204) | 2 (1–3) | 88 (43.1) | 34 (16.7) | 42 (20.6) | 24 (11.8) | 16 (7.8) |
| j) (n = 206) | 2 (1–3) | 69 (33.5) | 39 (18.9) | 58 (28.2) | 32 (15.5) | 8 (3.9) |
| 1. (n = 204) | 3 (2–4) | 29 (14.2) | 41 (20.1) | 69 (33.8) | 43 (21.1) | 22 (10.8) |
| 1. (n = 204) | 2.5 (1–3) | 56 (27.5) | 46 (22.5) | 54 (26.5) | 34 (16.7) | 14 (6.9) |
| 1. (n = 205) | 3 (1–4) | 56 (27.3) | 38 (18.5) | 59 (28.8) | 31 (15.1) | 21 (10.2) |
| 1. (n = 202) | 3 (2–4) | 28 (13.9) | 39 (19.3) | 59 (29.2) | 44 (21.8) | 32 (15.8) |
| 1. (n = 212) | 3 (2–5) | 31 (14.6) | 28 (13.2) | 52 (24.5) | 48 (22.6) | 53 (25.0) |
| 1. (n = 205) | 2 (1–3) | 85 (41.5) | 41 (20.0) | 42 (20.5) | 19 (9.3) | 18 (8.8) |
| 1. (n = 206) | 2 (1–3) | 97 (47.1) | 28 (13.6) | 43 (20.9) | 23 (11.2) | 15 (7.3) |
| 1. (n = 202) | 2 (1–3) | 70 (34.7) | 33 (16.3) | 55 (27.2) | 25 (12.4) | 19 (9.4) |
| 1. (n = 206) | 3 (2–4) | 43 (20.9) | 35 (17.0) | 59 (28.6) | 47 (22.8) | 22 (10.7) |
| 1. (n = 215) | 2 (1–3) | 100 (46.5) | 28 (13.0) | 54 (25.1) | 21 (9.8) | 12 (5.6) |

Note. a) I feel fit, b) I do not feel able to do much physically, c) I feel very active, d) I want to do lots of fun things, e) I feel tired, f) I think I do a lot in a day, g) I am able to concentrate on what I am doing, h) I can do a lot physically (good physical stamina), i) I am stressed about having to do things, J) I think I get very little done in a day, k) I feel rested, l) It takes a lot of effort to concentrate, m) I feel out of shape physically, n) I have lots of plans, o) I get tired easily, p) I do not get much done, q) I do not feel like doing anything, r) My thoughts wander easily, s) I feel in excellent shape physically, t) I have difficulty concentrating or focusing my attention

Table 3.2. Additional data: Distribution of Multidimensional Fatigue Inventory (MFI) scores among the PCC respondents based on age, sex, hospitalization unit, and LOS after 4-6 months of SARS-CoV-2 infection.

| Age category (years) | | *P-values* |
| --- | --- | --- |
|  |  | *.12*** |
|  | Median (IQR 1–3) |  |
| 18–64 years (n = 64) | 56.5 (50.2–63) |  |
| 65–74 years (n = 79) | 56 (52–59) |  |
| 75 and older (n = 77) | 54 (48.5–59) |  |
| Gender | | *.36**** |
| Women (n = 91) | 55 (48–60) |  |
| Men (n = 125) | 56 (51–59) |  |
| Hospitalization unit | | *.20**** |
| ICU (n = 36) | 54 (49.2–57) |  |
| Other units (n = 184) | 56 (50.2–60) |  |
| Length of stay | | *.71** |
| < 11days (n = 104) | 56 (50–59) |  |
| ≥ 11 days (n = 115) | 55 (50–60) |  |

Note. * = Mann–Whitney test;** = Kruskal–Wallis test; *** = Chi-squared test; ICU = Intensive care unit

Table 4.1. Additional/detailed data: Distribution of concentration, attention, and memory disorders with the Q3PC after 4-6 months of SARS-CoV-2 infection.

| Scale items | Never  n (%) | Rarely  n (%) | Sometimes  n (%) | Often  n (%) | Very Often  n (%) |
| --- | --- | --- | --- | --- | --- |
| Do you suffer from memory loss? (n = 212) | 62 (29.2) | 40 (18.9) | 69 (32.5) | 30 (14.2) | 11 (5.2) |
| Do you feel a slowness of reasoning in planning your activities to solve a problem? (n = 211) | 75 (35.5) | 36 (17.1) | 66 (31.3) | 22 (10.4) | 12 (5.7) |
| Do you find it difficult to concentrate or focus your attention (e.g., reading, in conversation)? (n = 215) | 100 (46.5) | 28 (13) | 54 (25.1) | 21 (9.8) | 12 (5.6) |

Table 4.2. Additional/detailed data: Distribution of concentration, attention, and memory disorder scores on the Q3PC scale after 4-6 months of SARS-CoV-2 infection.

|  | Median (IQR 1–3) | *P-values* |
| --- | --- | --- |
| Age category (n = 217) |  |  |
| 18–64 years (n = 64) | 4.5 (1.2–7) | *.07*** |
| 65–74 years (n = 78) | 3 (0–6) |  |
| 75 years and older (n = 75) | 3 (1–6) |  |
| Gender (n = 241) |  | *.97**** |
| Women (n = 90) | 3.9 (1–6) |  |
| Men (n = 123) | 3.8 (1–6) |  |
| Hospitalization unit (n = 217) |  |  |
| ICU (n = 36) | 3 (3–6) | *.20**** |
| Other units (n = 181) | 4 (1–6) |  |
| Length of stay (n = 216) |  |  |
| < 11 days (n = 103) | 3 (1–5) | *.03** |
| ≥ 11 days (n = 113) | 4 (1–6) |  |

Note. * = Mann–Whitney test;** = Kruskal–Wallis test; *** = Chi-squared test; ICU = Intensive care unit

Table 5.1. Additional/detailed data: Distribution of PHQ-4 among infected participants during hospitalization and 4-6 months after infection.

| **Scale items** | Never  n (%) | Some days  n (%) | > 50% of days  n (%) | Almost every day  n (%) | Median  Scores  (IQR 1–3) |
| --- | --- | --- | --- | --- | --- |
| **Feel nervous, anxious, or on edge** | | | | | |
| During hospitalization | 131 (53.5) | 58 (23.7) | 21 (8.6) | 35 (14.3) | 0 (0–1) |
| Four months after COVID-19 infection | 154 (62.9) | 60 (24.6) | 19 (7.8) | 12 (4.9) | 0 (0–1) |
| **Have little interest in or pleasure doing things** | | | | | |
| During hospitalization | 128 (52.2) | 50 (20.4) | 33 (13.5) | 34 (13.9) | 0 (0–2) |
| Four months after COVID-19 infection | 156 (63.7) | 59 (24.1) | 14 (5.7) | 16 (6.5) | 0 (0–1) |
| **Unable to stop or control my worries** | | | | | |
| During hospitalization | 147 (60.0) | 46 (18.8) | 29 (11.8) | 23 (9.4) | 0 (0–1) |
| Four months after COVID-19 infection | 177 (72.2) | 47 (19.2) | 10 (4.1) | 11 (4.5) | 0 (0–1) |
| **Feel demoralized, depressed, or hopeless** | | | | | |
| During hospitalization | 143 (58.4) | 48 (19.6) | 22 (9) | 32 (13.1) | 0 (0–1) |
| Four months after COVID-19 infection | 167 (68.2) | 48 (19.6) | 15 (6.1) | 15 (6.1) | 0 (0–1) |

Table 5.2. Additional/detailed data: Distribution of PHQ-4 by age category, length of stay, sex, and hospitalization unit during hospitalization and after 4-6 months hospital discharge.

|  | During hospitalization | *P-values* | After four months | *P-values* |
| --- | --- | --- | --- | --- |
|  | Median (IQR 1–3) | During Hosp | Median (IQR 1–3) | After 4 months |
| **Age category (n = 238)** |  | *.27*** |  | *.82*** |
| 18–64 years (n = 64) | 2 (0–6) |  | 1 (0–3) |  |
| 65–74 years (n = 87) | 1 (0–4) |  | 0 (0–2) |  |
| 75 years and older (n = 87) | 2 (0–5) |  | 1 (0–3) |  |
| **Gender (n = 241)** |  | *.03**** |  | *.03**** |
| Women (n = 100) | 2 (0–8) |  | 1 (0–4) |  |
| Men (n = 141) | 2 (0–4) |  | 0 (0–3) |  |
| **Hospitalization unit (n = 245)** |  | *< .01**** |  | *.04**** |
| ICU (n = 41) | 4 (1.5–8) |  | 2 (0–4) |  |
| Other units (n = 204) | 2 (0–4) |  | 0 (0–3) |  |
| **Length of stay(n = 244)** |  | *.02 ** |  | *.03** |
| < 11 days (n = 120) | 2 (0–4) |  | 1 (0–2) |  |
| ≥ 11 days (n = 124) | 2 (0–6) |  | 1 (0–4) |  |

Note. * = Mann–Whitney test;** = Kruskal–Wallis test; *** = Chi-squared test; ICU = Intensive care unit

Table 6.1. Additional/detailed data: Score distributions of PTSD-20 PCL-5 scale items 4-6 months after COVID-19 infection.

| Items | Median  (IQR 1–3) | Not at all = 0  n (%) | A little = 1  n (%) | Sometimes = 2  n (%) | Often = 3  n (%) | Very often = 4  n (%) |
| --- | --- | --- | --- | --- | --- | --- |
| a) (n = 209) | 1 (0–2) | 89 (42.6) | 39 (18.7) | 47 (22.5) | 25 (12) | 9 (4.3) |
| b) (n = 208) | 0 (0–1) | 128 (61.5) | 37 (17.8) | 24 (11.5) | 12 (5.8) | 7 (3.4) |
| c) (n = 207) | 0 (0–1) | 124 (59.9) | 35 (16.9) | 30 (14.5) | 14 (6.8) | 4 (1.9) |
| d) (n = 208) | 0 (0–2) | 106 (51) | 40 (19.2) | 38 (18.3) | 15 (7.2) | 9 (4.3) |
| e) (n = 213) | 0 (0–2) | 117 (54.9) | 35 (16.4) | 35 (16.4) | 17 (8) | 9 (4.2) |
| f) a (n = 206) | 0 (0–1) | 126 (61.2) | 31 (15) | 31 (15) | 13 (6.3) | 5 (2.4) |
| f) b (n = 206) | 0 (0–1) | 126 (61.2) | 31 (15) | 31 (15) | 13 (6.3) | 5 (2.4) |
| g) (n = 209) | 0 (0–2) | 115 (55) | 36 (17.2) | 37 (17.7) | 10 (4.8) | 11 (5.3) |
| h) (n = 206) | 0 (0–1) | 143 (69.4) | 26 (12.6) | 21 (10.2) | 7 (3.4) | 9 (4.4) |
| i) (n = 208) | 0 (0–1) | 129 (62) | 37 (17.8) | 23 (9.4) | 12 (5.8) | 7 (3.4) |
| J) (n = 209) | 1 (0–2) | 99 (47.4) | 43 (20.6) | 37 (17.7) | 17 (8.1) | 13 (6.2) |
| k) (n = 210) | 0 (0–2) | 122 (58.1) | 33 (15.7) | 37 (17.6) | 9 (4.3) | 9 (4.3) |
| l) (n = 209) | 0 (0–1) | 149 (71.3) | 24 (11.5) | 21 (10) | 10 (4.8) | 5 (2.4) |
| m) (n = 206) | 0 (0–1.2) | 113 (54.9) | 42 (20.4) | 26 (12.6) | 15 (7.3) | 10 (4.9) |
| n) (n = 205) | 0 (0–0) | 170 (82.9) | 11 (5.4) | 16 (7.8) | 4 (2) | 4 (2) |
| o) (n = 206) | 0 (0–2) | 107 (51.9) | 44 (21.4) | 30 (14.6) | 19 (9.2) | 6 (2.9) |
| p) (n = 211) | 0 (0–1) | 127 (60.2) | 42 (19.9) | 23 (10.9) | 13 (6.2) | 6 (2.8) |
| q) (n = 212) | 1 (0–2) | 91 (42.9) | 33 (15.6) | 47 (22.2) | 24 (11.3) | 17 (8) |
| r) (n = 212) | 1 (0–3) | 72 (34) | 41 (19.3) | 41 19.3) | 31 (14.6) | 27 (12.7) |

Note. In the past month, in what way have you experienced the following: a) Repeated, distressing, involuntary memories of the stressful experience, b) Repeated, distressing dreams of the stressful experience, c) Suddenly feeling or acting as if you were going through the stressful experience again, d) Feeling upset when something reminds you of the experience, e) Reacting physically when something reminds you of the stressful experience, f) a- Avoiding memories, thoughts, and feelings related to the event, f) b- Avoiding people, things that remind you of the stressful experience g) Having difficulty remembering important elements of the stressful experience, h) Having negative beliefs about yourself, others, or the world, i) Having intense negative feelings such as fear, horror, anger, guilt, or shame, j) Losing interest in activities you used to enjoy, k) Feeling distant or disconnected from others, l) Being irritable, (k) Feeling distant or disconnected from others, (l) Feeling irritable, having angry outbursts, or acting aggressively, (n) Taking reckless risks or engaging in behaviors that could put you in danger, (o) Being "overly alert", vigilant, or on guard, (p) Being easily startled, (q) Having trouble concentrating, (r) Having trouble getting or staying asleep (PCL-5) (44).

Table 6.2. Additional/detailed data: Score distribution of PTSD-20 PCL-5 scale items according to age category, sex, hospitalization unit, and LOS after 4-6 months of SARS-CoV-2 infection.

| Age category | | *P-values* |
| --- | --- | --- |
|  |  | *.06*** |
|  | Median (IQR 1–3) |  |
| 18-64 years (n = 64) | 13.5 (6–31.7) |  |
| 65-74 years (n = 78) | 13.2 (3–17.2) |  |
| 75 years and older (n = 73) | 16.2 (3.5–25.5) |  |
| Gender | | *.35**** |
| Women (n = 87) | 17.5 (4–26) |  |
| Men (n = 124) | 14.7 (4–21.7) |  |
| Hospitalization Unit | | *.18**** |
| ICU (n = 36) | 20.5 (5–31.5) |  |
| Other units (n = 179) | 14.7 (4–21) |  |
| Length of Stay | | *.01** |
| < 11days (n = 105) | 12.7 (4–19.5) |  |
| ≥ 11 days (n = 109) | 18.7 (4–30) |  |

Note. * = Mann–Whitney test;** = Kruskal–Wallis test; *** = Chi-squared test; ICU = Intensive care unit

**Table 7*.*** Multivariate linear regression analysis of the number of persistent PCC symptoms and PHQ-4, Q3PC, and PCL-5 scores, adjusted for age, sex, and LOS, predicted MFI scores in the Valais Hospitals during the second and third waves of COVID-19 pandemic in 2020 and 2021 (n=245).

| Variables | | β | SE | Explanatory (β) | *t* test (*df*) | Significance (*P* value) | 95% CI |
| --- | --- | --- | --- | --- | --- | --- | --- |
| Intercept | | 52.536 | 1.236 |  | 42.519 | <.01 | 50.100 to 54.972 |
| PCC^a^ symptoms | | −.314 | .293 | −.084 | −1.073 (4) | .28 | −.892 to .263 |
| PHQ4^b^ | | −.633 | .293 | −.180 | −2.162 (4) | .03 | −1.211 to −.056 |
| Q3PC^c^ | | .642 | .274 | .202 | 2.344 (4) | .02 | .102 to 1.181 |
| PCL-5^d^ | | .108 | .072 | .152 | 1.502 (4) | .13 | −.034 to .249 |
| **Adjustment** | | | | | | | |
|  | Intercept | 57.091 | 5.384 |  | 10.605 | <.01 | 46.474 to 67.708 |
|  | PCC symptoms | −.259 | .298 | −.069 | −.871 (7) | .38 | −.847 to .328 |
|  | PHQ4 | −.545 | .295 | −.160 | −1.847 (7) | .07 | −1.127 to .037 |
|  | Q3PC | .646 | .273 | .203^e^ | 2.362 (7) | .02 | .107 to 1.185 |
|  | PCL-5 | .108 | .072 | .153^e^ | 1.497 (7) | .14 | −.034 to .250 |
|  | Gender | 1.562 | 1.517 | .072 | 1.030 (7) | .30 | −1.430 to 4.555 |
|  | Age | −.071 | .066 | −.078 | -1.074 (7) | .28 | −.201 to .059 |
|  | LOS | −1.669 | 1.556 | −.079 | −1.072 (7) | .28 | −4.738 to 1.401 |

^a^PCC: post-COVID conditions.

^b^PHQ4: patient health questionnaire-4.

^c^Q3PC: brief memory screening scale.

^d^PCL-5: posttraumatic stress disorders scale.

^e^Contributing to predicting MFI scores.

^f^LOS: length of stay.
